# The University of Michigan

**Published:** 1855-03

**Authors:** 


*The University of Michigan.*—We never mention this school without anticipating a sore-headed rejoinder. The report of the Board of Visitors is just out, however, and we make a few extracts by way of improvement on our previous articles on government schools.

The original plan of branch institutions is re-recommended and the consequences of their neglect thus stated:

"By referring to the history of the University, we find that, during the existence of its branches, its growth was quite healthy, in that its students were numerous, and well prepared to enter on a higher course of studies.

"Their abolishment, whatever may have been the causes, has proved a serious detriment to the University proper, and your Board would most earnestly suggest their reëstablishment as soon as possible.

"If the Honorable, the Legislature, deem it advisable to appropriate any funds for the University, at its coming session, allow us to suggest that it be for the branches of the University, in their upbuilding and perpetuation.

"They are needed to complete the idea of the wise men, by whose efforts and under whose guidance the University of Michigan was established. That idea is embodied in our constitution and statutory provisions, which require their existence.

"In absence of branches, sectarian institutions of various grades have been introduced to supply their place. And from this cause, there has arisen a demand for Professors in the University, of various beliefs, members of different schools of theology.

"The University of Michigan is a State Institution, for the people, as such, and not for any one, two, or more sects. And, in the opinion of your Board, the question should never be asked, 'of what denomination is he?' in reference to any candidate for Professorship. Ability to instruct, connected with a known moral character, are qualifications which should recommend their possessor to the attention of the Regents.

"These qualifications may be possessed by those who are, by profession, clergymen. At the same time, we suppose that, in accepting such a situation, a clergyman lays aside his robes, *pro tempore*, at least. Expediency, order, decency—a common respect for the citizens of a State, of all beliefs, would seem to require this, in those who occupy prominent positions in a State University, although it would be different in a sectarian Institution.

"Prudence would seem to dictate, that one occupying a Professor's chair would forego, as he should, the popular assemblage, as listeners to his opinions. In his situation he may be a partisan and a citizen, and enjoy his rights as such. But the nature of his position is such, that he is not called upon, while occupying it, to discharge those duties which he might well discharge did he not hold it. Ambition for office, for political or clerical distinction, can never be consistently realized by a Professor of the University of Michigan, as such.

"The condition and state of the University require that the members of its Faculty should not be candidates for office, nor public partisans, and also when they become such, their immediate resignation or removal. We are quite sure that the interests of the University have been injured by the neglect of duty on the part of some of the members of the Faculty, in endeavoring to aggrandize self; and if there be no other way to remedy this evil, we would suggest the passage of an additional section to act of April 8, 1851, checking it at once by legislation."

We once had occasion to review somewhat sharply an address by Prof. J. Adams Allen, on the "Medical Platform." Dr. Allen has since been removed from his professorship of Materia Medica. We willingly do him the justice to publish the following comments of the Board on his removal:

"The removal of Prof. J. Adams Allen, M. D., from the chair of 'Therapeutics, Materia Medica, and Pathology,' which he had hitherto filled with credit to himself, and with honor to the University, has caused some injurious reports to be circulated, which an explanation of the true cause of removal may, perhaps, refute.

"As far as this Board can determine, personal feeling and prejudice contributed to this action of the Regents, and not a want of capacity to discharge his duties, on the part of Dr. Allen."

The Detroit Free Press comments as follows on the Report of the Board of Visitors:

"Equally just are the remarks of the Board respecting the professorships in the University being used as stepping-stones to political honors and ecclesiastical preferments; and the rebuke to any gentlemen of the Faculty who may have entered the arena of politics and addressed partizan assemblages, is not the less severe for being moderately administered. It is notorious that a portion of the Faculty were active politicians in the late canvass. The Board 'most earnestly protest against such conduct, deeming it worthy of censure, and, if persisted in, cause of immediate removal.' 'We are quite sure,' the Board add, 'the interests of the University have been injured by neglect of duty on the part of some of the members of the Faculty, in endeavoring to aggrandize self; and if there be no other way to remedy this evil, we would suggest the passage of an additional section to the act of April 8, 1851, checking it at once by legislation.'"

We must place the Michigan University and the Smithsonian Institution in the same category. Give us — here in free America — private enterprise, with the stimulus of private success and reputation, and we will stake them against any school paralyzed by the "anæsthetic influence of government pap."

The deduction to be drawn from these statements of the Board is simply a confirmation of the objections raised long ago by us to government schools.

A medical school should recognize no higher power than the medical profession. But under the system to which we object there is a power behind the throne. Admitting as we do, most cordially, that the medical faculty of the Michigan University is mostly made up of men of zeal and attainment, amply qualified as teachers, it is nevertheless true that above these men are others, destitute of all attachment or veneration for medical science, who have the power to control the associations if not the acts of the medical professors.

The Legislature of Michigan has recently passed an act requiring the Board of Regents to establish a Chair of Homœopathy. This new addition to the advantages of the school is to possess all the rights and privileges of any professor. Is not this a "confirmation strong as Holy Writ" of our prophecies of evil? In what other of the medical colleges of our land could this keen insult to the profession have occurred? What Council or Board of Trustees would compel their Faculty to such an association? And what Faculty would for a moment endure it?

And yet what are the Ann Arbor professors to do? The first impulse is doubtless to resign. Perhaps the voice of the profession would require it. But in event of their resignation what becomes of all their hopes of a great medical reform commencing at Ann Arbor? Who is there, knowing the proclivities of President (or Chancellor!) Tappan, that can doubt that he would, if possible, reorganize an exclusive homœopathic faculty? To resign is to give up a pleasant residence, a decent salary, and to abandon their cherished school to the control of quacks. To remain is to submit to an insult degrading beyond measure, and to suffer under the consciousness that next year the eclectics will be entitled to a professorship.

While we feel that this discussion, which has excited so much unpleasant feeling, has in its termination justified all that we have uttered, and given to us an ample triumph, we look upon the fulfillment of our predictions as a calamity which we ourselves would have labored strenuously to avert, and which subjects men whom we honor and respect, to a most painful alternative. H.

